# Assessment of genetic and functional diversity of phosphate solubilizing fluorescent pseudomonads isolated from rhizospheric soil

**DOI:** 10.1186/1471-2180-8-230

**Published:** 2008-12-20

**Authors:** Popavath Ravindra Naik, Gurusamy Raman, Kannan Badri Narayanan, Natarajan Sakthivel

**Affiliations:** 1 Department of Biotechnology, Pondicherry University, Kalapet, Pondicherry 605014, India

## Abstract

**Background:**

Phosphorus is an essential macronutrient for the growth of plants. However, in most soils a large portion of phosphorus becomes insoluble and therefore, unavailable to plants. Knowledge on biodiversity of phosphate-solubilizing fluorescent pseudomonads is essential to understand their ecological role and their utilization in sustainable agriculture.

**Results:**

Of 443 fluorescent pseudomonad strains tested, 80 strains (18%) showed positive for the solubilization of tri-calcium phosphate (Ca_3_(PO_4_)_2_) by the formation of visible dissolution halos on Pikovskaya's agar. These phosphate solubilizing strains showed high variability in utilizing various carbon sources. Numerical taxonomy of the phosphate solubilizing strains based on their carbon source utilization profiles resulted into three major phenons at a 0.76 similarity coefficient level. Genotypic analyses of strains by BOX (bacterial repetitive BOX element)-polymerase chain reaction (PCR) resulted into three distinct genomic clusters and 26 distinct BOX profiles at a 80% similarity level. On the basis of phenotypic characterization and *16S rRNA *gene phylogenetic analyses strains were identified as *Pseudomonas aeruginosa, P. mosselii, P. monteilii, P. plecoglossicida, P. putida, P. fulva *and *P. fluorescens*. These phosphate solubilizing strains also showed the production of plant growth promoting enzymes, hormones and exhibited antagonism against phytopathogenic fungi that attack on various crops. Gene specific primers have identified the putative antibiotic producing strains. These putative strains were grown in fermentation media and production of antibiotics was confirmed by thin layer chromatography (TLC) and high performance liquid chromatography (HPLC).

**Conclusion:**

Present study revealed a high degree of functional and genetic diversity among the phosphate solubilizing fluorescent pseudomonad bacteria. Due to their innate potential of producing an array of plant growth promoting enzymes, hormones and antifungal metabolites these phosphate solubilizing strains are considered to play a vital role in plant growth promotion, disease suppression and subsequent enhancement of yield.

## Background

Phosphorous deficiency is a major constraint to crop production. Plants absorb only inorganic form of phosphorous and the level of inorganic phosphorus is very low in the soil because most of the phosphorous is present as insoluble forms. Agricultural soils possess considerable accumulation of phosphorous due to the regular excessive applications of chemical fertilizers. The overuse of chemicals and chemical fertilizers has led to the lethal consequences to useful arthropods and other beneficial microbes as well as led to soil pollution. A large proportion of these fertilizers added to the soil are also converted to insoluble form and become unavailable to plants [1].

Microorganisms have the ability to solubilize the insoluble phosphates and maintain the soil health and quality [2]. Bacteria use several direct and indirect mechanisms of action to improve plant growth and health. Mechanisms such as phosphate solubilization [3], aminocyclopropane-1-carboxylate (ACC) deaminase [4], nitrogen cycle [5] and phytohormone production [6] are considered as popular mechanisms. Beneficial effects of phosphate solubilizing bacteria to crops have been documented well [1,7,8]. It is believed that microbial solubilization of phosphate in soil was correlated with the ability of microbes in producing selected organic acids and or extracellular polysaccharides [3,9]. This hypothesis has been corroborated by cloning pyrroloquinoline (PQQ) synthase [10-12] and *gab*Y genes involved in gluconic acid production. Gluconic acid is the principal organic acid produced due to direct oxidation of glucose by *Pseudomonas *which was found to be involved in phosphate solubilization [13].

Among phosphate solubilizing bacteria, fluorescent pseudomonads that colonize aggressively the plant roots have been considered as an important group of bacteria due to their biofertilizing and biocontrol properties. In many occasions, plant growth promoting rhizobacteria often exhibit the production of antimicrobial metabolites, which take part in suppression of diseases caused by soil-borne phytopathogens [6,14,15] as well as involve in the induction of systemic resistance against insects and nematodes [16-18]. Selective strains of fluorescent pseudomonad bacteria have also been reported for biodegradation of agricultural pollutants [15,19] as well as for weed control in agricultural fields [20-22]. These bacteria have been considered as an important bioinoculants due to their innate potential to produce plant growth promoting hormones and enzymes [6,16,23-25]. Though the bacteria belonging to *Mesorhizobium, Rhizobium, Klebsiella, Acinetobacter, Enterobacter, Erwinia, Achrobacter, Micrococcus, Aerobacter *and *Bacillus *have been reported as phosphate solubilizers, strains belong to pseudomonads are considered as efficient phosphate solubilizers [26].

Rice and banana are most important food crops of the world and are widely grown in developing countries. Fungal pathogens are important production constraints of rice, banana and other crops. Indiscriminate use of fungicides in agriculture is known to be hazardous to the environment and lethal toward other beneficial organisms; it can lead to the development of resistance against target organisms. Microbial solubilization of phosphorus, biodegradaton of chemical pollutants and biocontrol of plant pathogens are cost-effective, novel biological restoration and ecofriendly techniques. Considering the multiple applications of phosphate solubilizing fluorescent pseudomonads it is essential to study their diversity, which will be useful in designing strategies to use these native strains as bioinoculants for sustainable and organic agriculture without causing harm to the environment and farmers. The objective of the present investigation was to study the genetic and functional diversity of phosphate solubilizing fluorescent pseudomonads associated with rhizospheric soils of rice and banana by an array of *in vitro *assays, gene amplification techniques, fermentation methods and chromatographic analyses. Taxonomic affiliation of bacteria was done on the basis of *16S rRNA *gene similarity and molecular phylogenetic analyses.

## Methods

### Isolation of fluorescent pseudomonad strains

Rhizospheric soil samples of rice (*Oryza sativa *L.) and banana (*Musa *spp. AAA) were collected from agricultural fields located at Puducherry, India. Samples were stored at 4°C before being processed within 24 h of collection. The soil was sand clay loam and its characteristics were as follows; pH 7.3, 74 μg g^-1 ^nitrogen, 2.9 μg g^-1 ^phosphorus, 5.29 μg g^-1 ^potassium, 0.2 mOhms cm^-1 ^of electrical conductivity (EC). Isolation of fluorescent pseudomonad bacteria from rhizospheric soil was performed as described earlier [25]. Briefly, soil suspension was obtained by shaking 10 g of soil sample having roots with tightly adhering soil in 90 ml of 0.1 M MgSO_4_.7H_2_O buffer for 10 min at 180 rpm on a rotary shaker. Resulting suspensions were serially diluted and 0.1 ml aliquots of each dilution were spread onto King's medium B (KB) agar in triplicates. After incubation at 28°C for 2 days, fluorescent pseudomonad colonies from replicate plates were identified under UV light (366 nm). Purified single colonies were further streaked onto KB agar plates to obtain pure cultures. Stock cultures were made in Luria Bertani (LB) broth containing 50% (w/v) glycerol and stored at -86°C.

### Screening of phosphate solubilizers and estimation of phosphate solubilization

To detect the phosphate solubilizing bacteria, strains were streaked onto Pikovskaya's agar medium, which contains (per liter): 0.5 g yeast extract, 10 g dextrose, 5 g Ca_3_(PO_4_)_2_, 0.5 g (NH_4_)_2_SO_4_, 0.2 g KCl, 0.1 g MgSO_4_.7H_2_O, 0.0001 g MnSO_4_.H_2_O, 0.0001 g FeSO_4_.7H_2_O and 15 g agar. After 3 days of incubation at 28°C, strains that induced clear zone around the colonies were considered as positive [27]. Determination of phosphate solubilizing activity by the strains was carried out following standard method [28]. Briefly, cells were grown in liquid medium (pH 7.0) at 28°C up to 10 days and on 1, 3, 5, 7 and 10 day an aliquot of 5 ml was collected and cells were removed by centrifugation at 9,000 *g *for 20 min. Soluble free phosphate in culture supernatant was estimated from the absorbance values obtained using the calibration curve with KH_2_PO_4 _at 600 nm for each strain. Also, pH variation in Pikovskaya's medium during the growth of each strain was also observed.

### Phenotypic characterization of phosphate solubilizing bacteria

In order to determine the phenotypic diversity of 80 phosphate solubilizing bacteria characterization was done on the basis of fluorescence on King's B (KB) medium, levan formation, gelatin liquefaction, nitrate reduction and growth at 4°C and 42°C [29,30]. In order to identify fluorescence of the strains, overnight grown cells on KB agar medium were visualized under UV light (366 nm). Gram's reaction was determined by the KOH technique [31]. Briefly, visible amount of overnight grown cells from agar plate was smeared (1–2 cm^2 ^area) onto glass slide containing loopful (3 mm) of 3% aqueous KOH solution. Gram negative strains were identified as viscous gel that string out along with the loop. To test the presence of cytochrome oxidase, bacterial culture grown for 24 h on nutrient agar (NA) supplemented with 1% glucose was used. A loopful cells were rubbed onto a filter paper impregnated with 1% (wt/vol) aqueous *N,N,N',N'*-tetra-methyl-p-phenylenediamine dihydrochloride solution. A change in the color of the cultures to deep purple within 10 s was registered as a positive result. Presence of arginine dihydrolase and levan sucrase was tested on Thornley's medium and NA supplemented with arginine and sucrose (20 g l^-1^), respectively [29].

Carbon utilization profiles were tested using Hicarbohydrate™ kit as descried by the manufacturer (Himedia Laboratories, Mumbai, India). Cells were grown in nutrient broth to reach density of 0.5 O.D. at 600 nm. An aliquot of 50 μl of this suspension was inoculated to each well of Hicarbohydrate™ kit, incubated at 30°C for 24 h and the results were registered according to the instructions of the manufacturer. The experiment was done with three replicates. On the basis of data derived from the carbon source utilization profiles, a matrix with binary code composing positive (1) and negative (0) values was made. SIMQUAL program was used to compute the symmetric matrix in the form of average taxonomic distances. Sequential, agglomerative, hierarchical and nested (SAHN) clustering was used for the cluster analyses [32]. Dendrogram was constructed from the similarity matrix by the unweighted pair group with mathematical averages (UPGMA) using NTSYS-pc2.02a (Exeter software, New York, USA) numerical taxonomy and multivariate analysis system.

### BOX-PCR based genotypic analysis

Bacteria were cultured in LB broth at 28°C for 18 h and the total genomic DNA was extracted as described [25]. The BOX-A1R (5'-CTACGGCAAGGCGACGCTGACG-3') primer for genotypic analysis [33] was synthesized by Integrated DNA Technologies Inc. (Coralville, IA, USA). PCR reaction (50 μl) contained 50 pM of primer, 50 ng of genomic DNA, 1× *Taq *DNA polymerase buffer, 1 U of *Taq *DNA polymerase (Promega, Madison, WI), 0.2 mM of each deoxynucleotide triphosphate (dNTP), and 1.5 mM MgCl_2_. Amplification was performed in a DNA thermal cycler (2400 cycler, Perkin Elmer International, Rotkreuz, Switzerland) programmed with an initial denaturation at 95°C for 7 min, 30 cycles at 94°C for 1 min, 53°C for 1 min, and 65°C for 8 min, with an extension at 65°C for 15 min. A 10 μl of PCR product was separated using 1.5% agarose gel stained with ethidium bromide in 1× tris acetate ethylenediaminetetraacetic acid (TAE). The image of the gel was digitized by using BIO-CAPT system (Vilber Lourmat, France) and stored as TIFF files. BOX-PCR band profiles were detected by QuantityOne program (Bio-Rad Laboratories, CA, USA). Computer assisted analysis of genomic fingerprints was performed by using the BIO-GENE software program v11.02 (Vilber Lourmat, France). Similarity matrices of whole densitometric curves of the gels tracks were calculated by using the Dice coefficient. Cluster analysis of similarity matrices was performed by the un-weighted pair group with mathematical average (UPGMA) algorithm.

### 16S rRNA gene amplification, sequencing and phylogenetic tree analysis

Amplification of *16S rRNA *gene was performed from the genomic DNA of strains using universal primers fD1 (5'-GAGTTTGATCCTGGCTCA-3') and rP2 (5'-ACGGCTACCTTGTTACGACTT-3') [34]. PCR cocktails (50 μl) contained 50 pM of primer, 50 ng of genomic DNA, 1× *Taq *DNA polymerase buffer, 1 U of *Taq *DNA polymerase (Promega, Madison, WI, USA), 0.2 mM of each dNTP, and 1.5 mM MgCl_2_. Amplification was performed in a DNA thermo cycler (2400 cycler, Perkin Elmer International, Rotkreuz, Switzerland) at 94°C for 3 min, followed by 30 cycles of 10 s at 94°C, 1 min at 56°C and 30 s at 72°C with an extension of 72°C for 5 min. A 5 μl aliquot of each amplification product was electrophoresed on a 0.7% agarose gel in 1× TAE buffer at 50 V for 45 min, stained with ethidium bromide and the PCR products were visualised with a UV transilluminator. PCR products were purified using Quick PCR purification column (Promega, Madison, USA). Purified PCR products were sequenced with automated DNA sequencer with specific primers using the facility at Macrogen Inc. (Seoul, Korea). To perform molecular phylogenetic analyses, reference sequences required for comparison were downloaded from the EMBL database using the site . All the sequences of *16S rRNA *were aligned using the multiple sequence alignment program CLUSTAL W [35]. The aligned sequences were then checked for gaps manually, arranged in a block of 600 bp in each row [36] and saved as molecular evolutionary genetics analysis (MEGA) format in software MEGA v3.0. The pair wise evolutionary distances were computed using the Kimura 2-parameter model [37]. To obtain the confidence values, the original data set was resampled 1000 times using the bootstrap analysis method. The bootstrapped data set was used directly for constructing the phylogenetic tree using the MEGA v3.0 program for calculating the multiple distance matrixes [38]. The multiple distance matrix obtained was then used to construct phylogenetic trees using neighbor-joining (NJ) method [39].

### Nucleotide sequence accession numbers

The nucleotide sequences of *16S rRNA *were deposited in GenBank. The accession numbers of the *16S rRNA *nucleotide sequences of the strains are presented in Additional file 1.

### Production of other plant growth promoting enzymes and hormones

#### Indole-3-acetic acid (IAA)

Production of IAA was determined following the standard method [40]. Briefly, overnight grown single colony was streaked onto LB medium agar, which contains (per liter): 10 g tryptone, 5 g yeast extract, 5 g NaCl, 15 g agar amended with 5 mM L-tryptophan, 0.06% sodium dodecyl sulphate and 1% glycerol. Plates were overlaid with sterile Whatman no. 1 filter paper (82 mm diameter) and bacterial strain was allowed to grow for 3 days at 28°C. After incubation, the paper was removed and treated with Salkowski's reagent [41] having the formulation of 2% of 0.5 M ferric chloride in 35% perchloric acid at room temperature for 60 min. In a Petri dish, the filter papers were saturated by soaking in Salkowski's reagent and the production of IAA was identified by the formation of a red halo on the paper immediately surrounding the colony.

#### Aminocyclopropane-1-carboxylate (ACC) deaminase

The ACC deaminase activity was determined as described earlier [16] on Dworkin and Foster (DF) minimal salts medium, which contains (per liter): 4 g KH_2_PO_4_, 6 g Na_2_HPO_4_, 0.2 g MgSO_4_.7H_2_O, 2 g glucose, 0.2 g; 2 g gluconic acid and 2 mg citric acid with trace element solution (1 mg FeSO_4_.7H_2_O, 10 μg H_3_BO_3_, 11.19 μg MnSO_4_.H_2_O, 124.6 μg ZnSO_4_.7H_2_O, 78.22 μg CuSO_4_.5H_2_O and 10 μg MoO_3_). Filter sterilized ACC solution (3 mM) was spread over the agar plates, allowed to dry for 10 min and inoculated with bacterial strains. Observation of the growth made after 2 days incubation at 28°C as described earlier [4].

#### Siderophore

Production of siderophore by the strains was determined using the FeCl_3 _test and the chrome azurol S agar assay. Briefly, inoculum (10 μl) of bacterial strains dropped onto the center of a CAS plate. After incubation at 25°C for 3 days, siderophore production was assessed on the basis of change in color of the medium from blue to orange [42-44].

### Production of fungal cell wall degrading enzymes

#### Protease

The protease activity was determined using skim milk agar medium, which contains (per liter): 5 g pancreatic digest of casein, 2.5 g yeast extract, 1 g glucose, 7% skim milk solution and 15 g of agar. Bacterial cells were spot inoculated and after 2 days incubation at 28°C proteolytic activity was identified by clear zone around the cells [45].

#### Chitinase

The chitinase activity of strains was tested on chitin agar medium, which contains (per liter): 1.62 g nutrient broth, 0.5 g NaCl, 6 g M9 salts, 8 g colloidal chitin and 15 g agar. Bacterial cells were spot inoculated and after 5 days incubation at 30°C, chitinase activity was identified by clear zone around the cells [17].

### Production of other beneficial enzymes

#### Cellulase

Strains were screened for cellulase production by plating onto M9 medium agar amended with 10 g of cellulose and 1.2 g of yeast extract per liter. After 8 days of incubation at 28°C, colonies surrounded by clear halos were considered positive for cellulase production [23].

#### Pectinase

Pectinase production was determined using M9 medium amended with 4.8 g of pectin per liter. After 2 days of incubation at 28°C, plates were flooded with 2 mol l^-1 ^HCl and strains surrounded by clear halos were considered positive for pectinase production [23].

#### Test for antagonism

Strains were tested for *in vitro *antagonism towards fungal pathogens by following standard co-inoculation technique on potato dextrose agar (PDA) [36,46]. Fungal phytopathogens, *Fusarium oxysporum *f. sp. *cubense *FOC (discoloration of banana), *Cylindrocladium floridanum *ATCC42971 (root necrosis of banana), *Cylindrocladium scoparium *ATCC46300 (root necrosis of banana), were kindly provided by J. M. Risede, UMR de pathologie végétale INRA-INH-Universite d'Angers, Beaucouze Cedex and CIRAD-FLHOR, Station de Neufchateâu, Sainte Marie, Capesterre-Belle-Eau, Guadeloupe, France. *Rhizoctonia solani *RSR1 (sheath blight of rice), *Magnaporthe grisea *MGS (blast of rice), *Sarocladium oryzae *SONS (sheath rot of rice), *Botrytis cinerea *BCTNAU (Blight of tobacco), *Macrophomina phaseolina *MPS (charcoal rot of ground nut), *Pestalotia theae *PTS (leaf spot of tea), *Colletotrichum falcatum *(red rot of sugarcane), *C. capsici *CPS (fruit rot of chili), *C. gleosporoides *CGS (Anthracnose of mango) were obtained from the Microbial Culture Collection (MCC), Department of Biotechnology, Pondicherry University, Puducherry. These phytopathogenic fungi are major production constraints of rice, banana and other crops and therefore, selected for screening antagonism. Briefly, bacterial plugs (6 mm diameter) were removed from a 48 h culture and were transferred to the center of PDA plates, which had been inoculated with fungal spore suspension (10^6 ^conidia ml^-1^). Assay plates were incubated at 28°C for 3 days and growth inhibition appeared around the bacterial plugs was measured. Assays were done with three replicates.

### Screening of putative antibiotic producing strains by polymerase chain reaction

Detection of the genes that encode for the production of antibiotics such as, 2,4-diacetylphloroglucinol (DAPG), phenazine-1-carboxylic acid (PCA), phenazine-1-carboxamide (PCN), pyrrolnitrin (PRN) and pyoluteorin (PLT) was done by PCR using gene-specific primers. Reference strains, *Pseudomonas fluorescens *Pf5, *P. fluorescens *2–79, *P. aureofaciens *30–84 (now considered as *P. chlororaphis*) and *P. aeruginosa *PAO1 were kindly supplied by Linda S. Thomashow, USDA, Washington State University, Pullman and *P. fluorescens *CHAO was kindly supplied by Geneviève Défago, Swiss Federal Institute of Technology, Zurich. Oligonucleotide primers were synthesized by Integrated DNA Technologies Inc. (Coralville, IA, USA). The primer sets and the amplification conditions for the screening of genes encoding antibiotics are listed in Table 1. PCR reaction (50 μl) contained 50 pM of each primer, 50 ng of genomic DNA, 1× *Taq *DNA polymerase buffer, 0.5 U of *Taq *DNA polymerase (Promega, Madison, WI, USA), 0.2 mM of each deoxynucleotide triphosphate, and 1.5 mM MgCl_2_. Amplification was performed in a DNA thermal cycler. A 5 μl aliquot of each amplified product was electrophoresed on a 0.7% agarose gel in 1× TAE buffer at 50 V for 45 min, stained with ethidium bromide and the PCR products were visualized with a UV transilluminator.

**Table 1:** Primers and amplification conditions for the different PCR based screening of genes that encode for antibiotics

Gene and primer set	Primer sequence	Reference	Amplification conditions
**PCA (C, D)** PCA2a PCA3b	5'-TGCCAAGCCTCGCT CCAAC-3' 5'-CGCGTTGTTCCTCGTTCAT-3'	77	Initial denaturation 94°C for 3 min, 30 cycles of 94°C for 60 s, 58°C for 45 s, and 72°C for 60 s. Final extension at 72°C for 10 min.
**PCA (X, Y)** 30–84/1 30–84/2	5'-CAGTTCATCCGGCGGGCTGCAG-3' 5'-CCCGTTTCAGTAAGTCTTCCATG- ATGCG-3'	77	Initial denaturation 94°C for 3 min, 30 cycles of 94°C for 60 s, 58°C for 45 s, and 72°C for 60 s, Final extension at 72°C for 10 min.
**PCN** PhzH-up PhzH-low	5'-CGCACGGATCCTTTCAGAATGT- TC-3' 5'-GCCACGCCAAGCTTCACGCTCA-3'	78	Initial denaturation 94°C for 30 s, 30 cycles of 94°C for 30 s, 64°C for 30 s, and 72°C for 7 min. Final extension at 72°C for 10 min.
**DAPG** Phl2a Phl2b	5'-GAGGACGTCGAAGACCACCA-3' 5'-ACC GCAGCATCGTGTATGAG-3'	79	Initial denaturation 94°C for 90 s, 35 cycles of 94°C for 35 s, 53°C for 30 s, and 72°C for 45 s. Final extension at 72°C for 10 min.
**PLT** PltBf PltBr	5'-CGGAGCATGGACCCCCAGC-3' 5'-GTGCCCGATATTGGTCTTGACCG- AG-3'	79	Initial denaturation 94°C for 2 min, 29 cycles of 94°C for 1 min, 58°C for 45 s, and 72°C for 1 min, Final extension at 72°C for 10 min.
**PRN** Prncf Prncr	5'-CCACAA GCCCGGCCAGGAGC-3' 5'-GAGAAGAGCGGGTCGATGAAG- CC-3'	79	Initial denaturation 94°C for 2 min, 30 cycles of 94°C for 1 min, 58°C for 45 s, and 72°C for 1 min. Final extension at72°C for 10 min.

### Production and quantification of antifungal metabolites and analytical methods

Antifungal metabolites were extracted as described [6,36,47]. Reference strains and antibiotic standards were kindly provided by Linda S. Thomashow, USDA, Washington State University, Pullman; and Geneviève Défago, Swiss Federal Institute of Technology, Zurich. Thin layer chromatography (TLC) was carried out on silica gel G60 plate (20 × 20 cm; 0.25 mm thick, Selecto Scientific, GA, USA). The plates were activated at 110°C for 30 min, cooled and spotted with ethanol solution containing standard antibiotic (0.5 μg) and 20 μl of the extract. Separation was performed using the solvent system: chloroform-methanol (9:1 vol/vol) for PCA and DAPG or chloroform-acetone (9:1 vol/vol) for PLT and PRN. The corresponding spots by PCA, and DAPG were detected by UV irradiation at 254 nm [48]. PLT spots were detected by spraying with an aqueous 0.5% (wt/vol) Fast Blue RR salt solution 0.5% (wt/vol) and the PRN spots were detected by spraying the TLC plates with 2% p-dimethylaminobenzaldehyde dissolved in the ethanol-sulfuric acid (1:1 vol/vol). Detection and quantitative determination of antibiotics was done by analytical HPLC (Shimadzu, Kyoto, Japan) as described [36]. Production of hydrogen cyanide (HCN) was determined by growing bacteria on KB agar medium supplemented with 4.4 g l^-1 ^glycine. A piece of filter paper impregnated with picric acid and sodium carbonate (0.5% and 2%, respectively) was placed in the lid of each Petri dish. Petri dish was sealed with parafilm and incubated at 28°C for 96 h. The production of cyanide was identified by the change in the color of filter paper from yellow to orange-brown [47].

## Results

### Isolation and screening of phosphate solubilizing bacteria and estimation of phosphate solubilization

**Figure 1:**
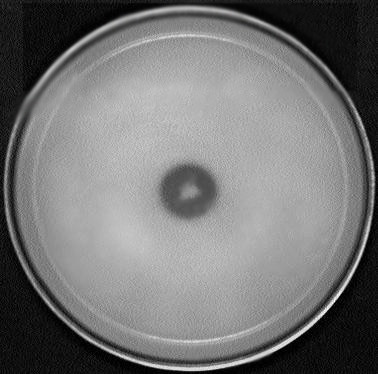
**Phosphate solubilization on Pikovskaya's agar medium by strain FPB9**.

Of the 443 strains, 80 strains (18%) produced phosphate solubilization on Pikovskaya's agar medium by inducing clear zones (Fig. 1). The solubilization of tri-calcium phosphate was estimated for all strains. Soluble phosphate was estimated to be 29 to 105 μg ml^-1 ^on 10 days of inoculation. A significant reduction in pH of Pikovskaya's liquid medium from pH 7.4 to pH 4.8 was observed on 10 days of incubation [See Additional file 2].

### Phenotypic characterization of phosphate solubilizing bacteria

**Figure 2:**
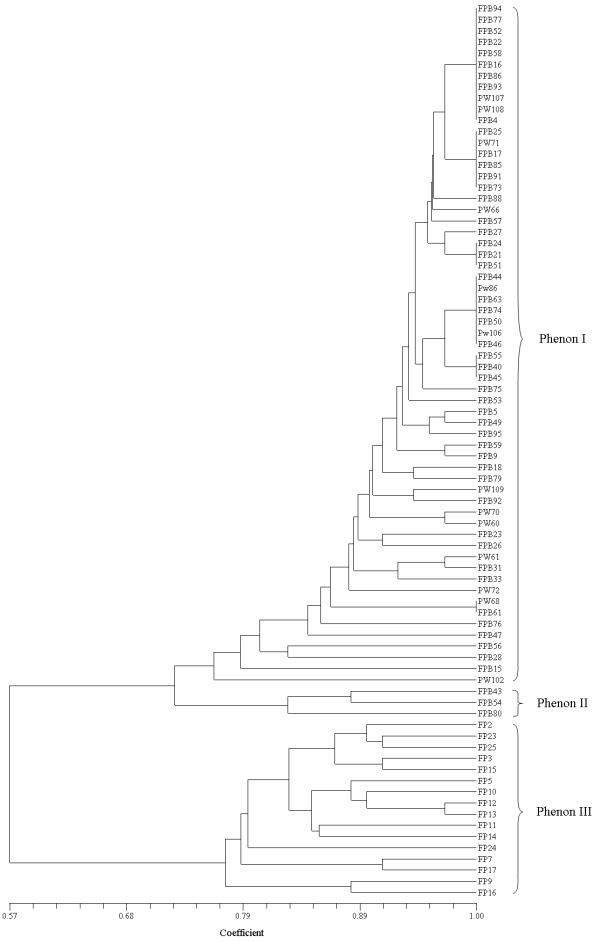
**Phenogram of 80 phosphate solubilizing fluorescent pseudomonads based on their carbon source utilization profiles**. The clustering was done using sequential, agglomerative, hierarchical and nested (SAHN) method. The pairwise coefficient of similarity (Dice) was used for clustering with the UPGMA algorithm using NTSYSpc2.02a software. The phenogram resulted into 3 major phenon at 0.76 smilarity coefficient. The experiment was done with three replicates.

Out of 443 fluorescent pseudomonad strains tested, 80 strains showed phosphate solubilization potential. These phosphate solubilizing strains were Gram-negative, motile, rod shaped and tested positive for cytochrome oxidase, arginine dihydrolase, strains showed variability for traits such as gelatin hydrolysis, levan production and growth at 4°C and 42°C. All phosphate solubilizing strains utilized dextrose, galactose, mannose and citrate but exhibited varying degree of utilization towards other carbon sources such as lactose, xylose, fructose, melibiose, L-arabinose, glycerol, ribose, α-methyl-D-mannoside, xylitol, esculin, D-arabinose, malonate, sorbose, trehalose, sorbitol, mannitol, adonitol and glucosamine. These strains did not utilize maltose, sucrose, inulin, salicin, dulcitol, inositol, α-methyl-D-gluconate, rhamnose, cellobiose, melazitose, xylitol and ONPG. Numerical analysis of phenotypic characteristics revealed a high degree of polymorphism. All phosphate solubilizing strains were grouped into 3 major phenons at 0.76 similarity coefficient level (Fig. 2). The similarity coefficient range among phosphate solubilizing strains was 0.57 to 1.00. Phenons I, II and III consist a total of 61, 3 and 16 strains, respectively.

### BOX-PCR based genotypic analysis

**Figure 3:**
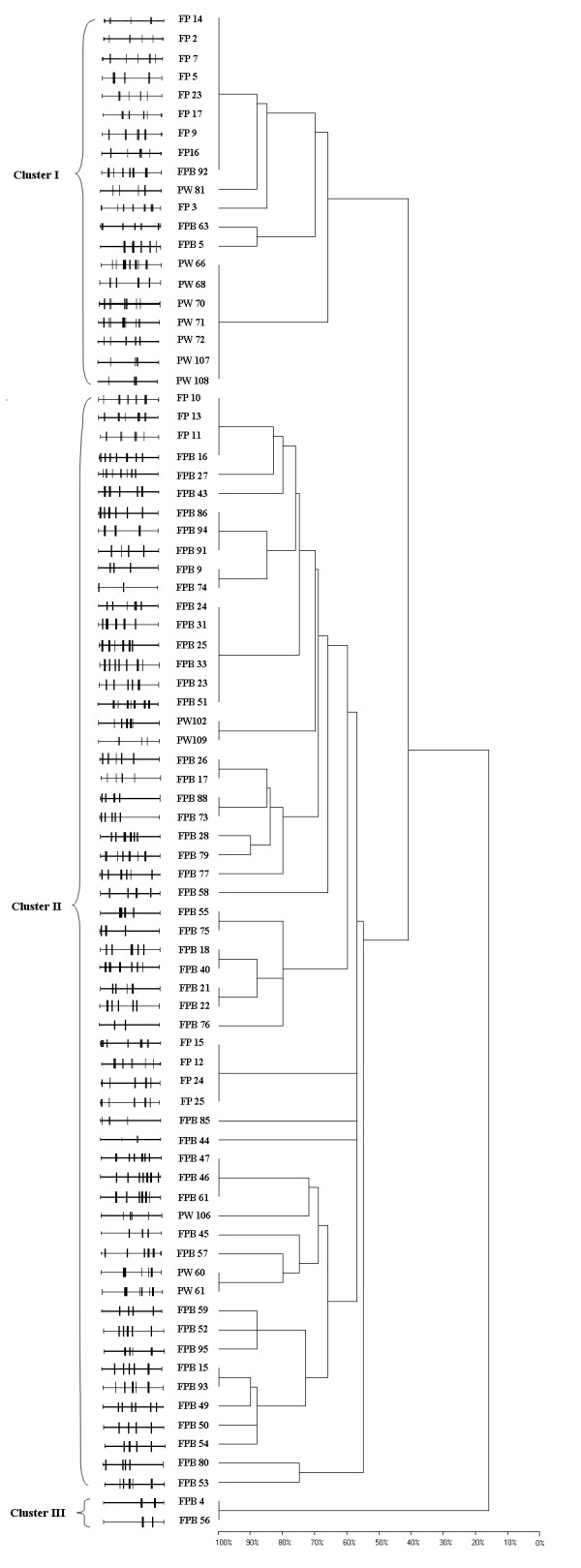
**Cluster analyses of BOX-PCR fingerprints showing the genotypic diversity of phosphate solubilizing fluorescent pseudomonads**. Dendrogram was obtained from the similarity coefficient (Dice) calculations and clustering was done using unweighted pair-grouping method based on arithmetic averages (UPGMA) algorithm using BIOGENE software v11.02. The dendrogram resulted into 3 major clusters and 26 distinct BOX profiles.

The cluster analysis based on the pair-wise coefficient similarity with UPGMA of BOX-PCR resulted into 3 distinct genomic clusters and at a 80% similarity coefficient generated 26 distinct BOX profiles (Fig. 3). A total of 20 strains were grouped into cluster I which shared 40% similarity with other strains. Cluster III consisted of 2 strains and a large number of strains grouped in cluster II comprising 58 strains. All the strains showed wide variations in fingerprinting pattern due to their high degree of genetic variability and distributed into different clusters. On the basis of these results, present study identified a high degree of genetic variability among different species of phosphate solubilizing fluorescent pseudomonads.

### 16S rRNA gene amplification, sequencing and phylogenetic tree analysis

**Figure 4:**
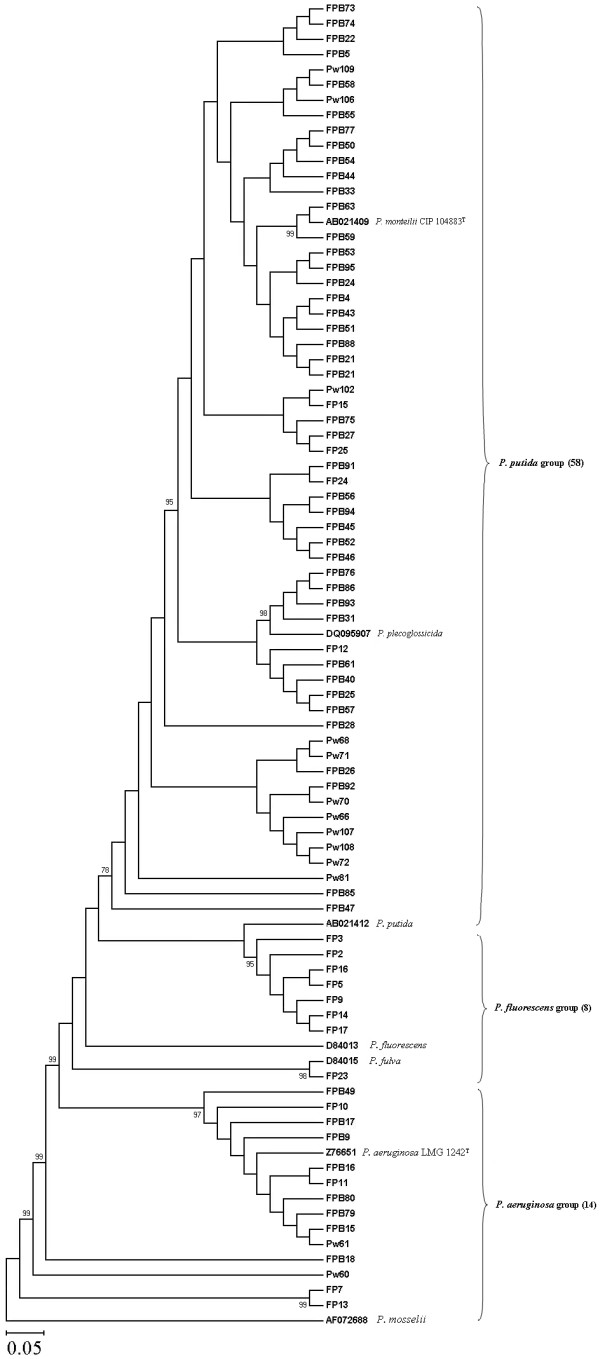
**Phylogenetic analyses of phosphate solubilizing strains of fluorescent pseudomonads based on the nucleotide sequence of *16S rRNA***. The multiple sequence alignment was done in CLUSTAL program. The pair-wise evolutionary distances were calculated using Kimura2-parameter model. The phylogenetic tree was constructed by neighbor-joining (NJ) method with 1000 replicates using bootstrap. A total of 7 reference fluorescent pseudomonad strains were used for the tree construction.

On the basis of phylogenetic analysis of *16S rRNA *gene (600 bp), species of pseudomonads such as *Pseudomonas monteilii, P. putida, P. plecoglossicida, P. fluorescens, P. fulva, P. mosselii *and *P. aeruginosa *were identified. Phylogenetic analyses of the 80 fluorescent pseudomonad strains based on the NJ method with 1000 bootstrap sampling were resulted into 3 major clusters (Fig. 4). Of the 80 strains, cluster I formed with 58 strains, cluster II formed with 8 strains and cluster III formed with 14 strains. A total of 36 strains belong to *P. monteilii*, 10 strains belong to *P. plecoglossicida*, 12 strains belong to *P. putida*, 7 strains belong to *P. fluorescens*, 1 strain belongs to *P. fulva*, 12 strains belong to *P. aeruginosa *and 2 strains belong to *P. mosselli *(See Additional file 1; Fig. 4).

### Production of plant growth promoting enzymes, hormones and fungal cell wall degrading enzymes

All the strains tested positive for the production of siderophore on the basis of change in colour of the CAS medium from blue to orange. Of the 80 phosphate solubilizing strains, 39 strains (49%) showed positive for the production of plant growth-promoting hormone, IAA. The ACC deaminase was observed in 13 strains (16%) by their growth on Dworking and Foster (DF) minimal medium containing ACC. Of the 80 strains, 22 strains (28%) showed proteolytic activity by inducing clear zones around the cells on skim milk agar medium, 7 strains (9%) showed chitinase activity by inducing clear zones around the cells on chitin agar medium, 33 strains (41%) produced pectinase and 25 strains (31%) produced cellulase [See Additional file 1].

### Test for antagonism

Of the 80 phosphate solubilizing strains, 33 strains (41%) showed antifungal activity towards phytopathogenic fungi used in the study [See Additional file 3]. Strains induced growth-free inhibition zones (diameter) ranging from 10 to 36 mm towards phytopathogenic fungi.

### Screening of putative antibiotic producing strains by PCR

Genomic DNA of strains when tested as templates using gene-specific primers, 12 strains (FPB16, FPB17, FPB51, FP2, FP3, FP5, FP7 FP10, FP14, Pw70, Pw71 and Pw72) amplified the DNA fragment of 745-bp of DAPG, 10 strains (FPB15, FPB16, FPB74, FPB75, FP2, FP3, FP5, FP7, FP14 and FP15) amplified the DNA fragment of 719-bp of PRN, 6 strains (FP2, FP3, FP5, FP7, FP9 and FP14) amplified DNA fragment of 779-bp of PLT and 6 strains (FPB18, FPB52, FPB75, Pw60, Pw61 and FP15) amplified DNA fragment of 1,150-bp of PCA (C, D).

### Production quantitative determination of antifungal metabolites

The putative antibiotic strains identified by PCR were subjected to fermentation and antifungal metabolites such as DAPG (yellowish white), PCA (greenish yellow), PRN (light yellow) and PLT (yellowish white) were detected in the production cultures. TLC and HPLC analyses confirmed the production of PCA, DAPG, PLT and PRN by the strains. The retardation factor (Rf) values were found to be 0.77 for DAPG, 0.53 for PCA, 0.80 for PRN, 0.50 for PLT as determined by co-migration with pure standards. Strains grown in fermentation cultures yielded PCA (20 to 1124 μg ml^-1^), DAPG (13 to 93 μg ml^-1^), PLT (3 to 9 μg ml^-1^) and PRN (3 to 41 μg ml^-1^). HPLC analyses of active fractions of antibiotics yielded similar retention time to that of the standard antibiotics. PCA was detected at 257 nm with a retention time 4.94 min, DAPG was detected at 270 nm with a retention time 10.77 min, PLT was detected at 255 nm with a retention time of 17.6 min and PRN was detected at 220 nm with a retention time of 27.5 min. Of the 80 strains, 20 strains (25%) produced HCN [See Additional file 1].

## Discussion

Phosphorus is considered as an essential macronutrient and a great portion of phosphorus from chemical fertilizers becomes insoluble by its conversion into calcium or magnesium salts in soils and become unavailable to plants. Soil microorganisms involve to transform the insoluble forms of phosphorus into soluble forms and thus influence the subsequent availability of phosphate to plant roots are considered essential [49,50]. Phosphate solubilizing microorganisms have been employed in agriculture and horticulture and have been considered very important due to their potential of ecological amelioration. It is believed that microbial mediated solubilization of insoluble phosphates in soil is through the release of organic acids microbial metabolites [51-53]. However, in addition to acid production, other mechanisms can cause phosphate solubilization [54]. Phosphate solubilization has been reported to depend on the structural complexicity and particle size of phosphates and the quantity of organic acid secreted by microbes [55].

Fluorescent pseudomonads often predominant among plant rhizosphere associated bacteria [24,56]. Plant growth promoting rhizobacteria are classified into two different groups such as strains that have the capability of synthesizing phytohormones and strains that have the ability to suppress the growth of phytopathogens [57]. Fluorescent pseudomonads enhance plant growth by improving soil nutrient status, producing plant growth hormones, enzymes and suppressing the growth of phytopathogenic fungi [56,58]. Plant growth promoting rhizobacterial types of fluorescent pseudomonads use one or more mechanisms of direct or indirect in improving plant growth. These mechanisms can probably be active simultaneously or sequentially at different stages of plant growth. This group of bacteria exhibits multiple functional traits such as solubilizing of inorganic phosphate and iron, production of vitamins, phytohormones and antimicrobial metabolites. They are capable of improving plant nutrients uptake, tolerance to stress, salinity, metal toxicity and pesticide. Fluorescent pseudomonad strains such as *P. fluorescens *NJ101 [14], *P. fluorescens *EM85 [59], *P. fluorescens *[60], *Pseudomonas *spp. [61,62], *P. chlororaphis*, *P. savastanoi*, *P. pickettii *[23] and *P. corrugata *[63] have been reported as phosphate solubilizers.

In the present investigation, out of 443 fluorescent pseudomonad strains screened, 80 strains have been identified as phosphate solubilizers. These strains were taxonomically described as different fluorescent pseudomonad species such as *P. monteilli*, *P. putida*, *P. plecoglossicida*, *P. fluoresens*, *P. fulva*, *P. monteilli *and *P. aeruginosa *on the basis of *16S rRNA *gene sequencing and subsequent molecular phylogeny analysis. Phenotypic analyses as well as *16S rRNA *and BOX-PCR based genotypic analyses revealed a high degree of diversity among phosphate solubilizing bacteria reported in this study. Significant decline in the pH of the culture medium by strains was observed during mineral phosphate solubilization, which suggested the microbial production of organic acids [64]. Although phosphate solubilization is not necessarily correlated with acidity, from the data present in this study relationship could be ascertained between the acidity of medium and the release of soluble phosphates. Estimation of phosphate solubilization of strains by other methods has been reported to be between 200 to 805 μg ml^-1 ^[65]. In an earlier study, *P. fluorescens *strain NJ-101 isolated from agricultural soil was reported to release 74.6 μg ml^-1 ^soluble phosphate from inorganic phosphate [14] and in the present study, up to 105.3 μg ml^-1 ^soluble phosphate was estimated. We have found that 49% of the strains produced IAA and 16% of the strains produced ACC deaminase. It is reported that the ACC deaminase producing bacteria increase root elongation and seed germination by lowering plant ethylene levels [24,66]. Specific strains of fluorescent pseudomonad bacteria indirectly influence the plant health by preventing the deleterious effects of phytopathogenic microorganisms through the production of antibiotics, cell wall degrading enzymes, HCN metabolite and siderophores [56,57]. Production of HCN by *P. fluorescens *CHAO was recognized as a biocontrol factor, against plant pathogenic fungi [67].

Production of antibiotics by fluorescent pseudomonads considered important in suppression of phytopathogens. Recently, *P. aeruginosa *PUPa3, a new strain from rice rhizosphere with potential for fungal antibiosis and biofertilizing traits has been identified from our laboratory [25]. All the strains reported in this study produced hydroxamate siderophores as evidenced on FeCl_3 _amended CAS agar medium, production of an array of phytohormones and antifungal metabolites. Microbial production of antibiotics, PCA (2 to 3 mg ml^-1^), DAPG (0.5 to 3 mg ml^-1^), PLT (1.5 to 2 μg ml^-1^) and PRN (0.54 mg ml^-1^) by biofertilizing and biocontrol strains have been reported in earlier studies [68-70]. In the present study the production of PCA (20 to 1124 μg ml^-1^), DAPG (13 to 93 μg ml^-1^), PLT (3 to 9 μg ml^-1^) and PRN (3 to 41 μg ml^-1^) by phosphate solubilizing fluorescent pseudomonads has been reported. Strain efficiency and variations in the fermentation conditions often result in an alteration in antibiotic production. Considering the quantity of antibiotics by plant growth promoting and biocontrol strains of fluorescent pseduomonads reported by other inverstigators [68-70], strains reported in this study may be considered as non-pathogenic to plants and antagonistic bacteria against phytopathogenic fungi. Strains reported in this study utilized several carbon sources as identified by Hi-carbohydrate™ kit test. Utilization of variety of carbon sources by the strains may play an important role in adapting to a variety of crop plants and soil types.

The IAA hormone is known to have dual role in influencing plant growth, by involving in the biocontrol together with glutathione-s-transferases in defense-related plant reactions and inhibits the germination of spore and growth of mycelium of different pathogenic fungi [71,72]. Martinez Noel et al. (2001) showed that the IAA supply to excised potato leaves reduced the severity of the disease provoked by *Phytophthora infestans *[73]. In present investigation, we have identified the spectrum of bacterial antagonism by measuring the inhibition zones of mycelial radial growth in plate assays. These antagonistic strains also showed production of IAA, fungal cell wall degrading enzymes, such as cellulases, proteases and chitinases which are known to be involved in antagonistic activity against phytopathogenic fungi and insects [74,75]. Selective microbial producers of chitinase are also reported to be the efficient phosphate solubilizers [76]. Phosphate solubilizing fluorescent pseudomonad strains reported in this study may solubilize insoluble compounds due to the excretion of organic acids. Production of antimicrobial metabolites and organic acids is essential to decrease soil pH, which plays a major role in solubilization of phosphates and other nutrients.

Characterization of phosphate solubilizing fluorescent pseudomonad bacteria is required to study their ecological role in soil. Fluorescent pseudomonad strains reported in this study with phosphate solubilization potential and ability to excrete phytohormones and antimicrobial metabolites may be used as plant growth promoting bacteria and biocontrol agents in sustainable agriculture.

## Conclusion

Phosphate solubilizing fluorescent pseudomonad bacterial strains with their multifunctional properties will attract more attention in the field of biofertilization and biological control. Present investigation revealed the microbial diversity of fluorescent pseudomonads with innate potential of mineralizing phosphate, plant growth promoting traits and biocontrol properties. Knowledge generated on biodiversity of phosphate solubilizing bacteria will be useful to design strategies to use these strains as inoculants in sustainable and organic agriculture.

## Authors' contributions

RN performed most experiments and crucial in writing the manuscript, GR performed estimation of phosphate solubilization; BN performed biochemical characterization, NS was vital in developing the key concepts and interpretation of results and approved draft of this manuscript.

## Supplementary Material

Additional file 1 Biochemical characterization and taxonomic identification of 80 phosphate solubilizing fluorescent pseudomonads. The data provided represent the taxonomic identification of strains on the basis of 16S rRNA nucleotide sequence based phylogenetic analysis, plant growth promoting traits (production of IAA, ACC deaminase and protease), plant growth affecting traits (production of pectinase and cellulase), biocontrol traits (production of chitinase, HCN and antagonism towards phytopathogenic fungi) and genotypic grouping on the basis of BOX-PCR fingerprint pattern. Click here for file

Additional file 2 Tricalcium phosphate solubilization by the strains of fluorescent pseudomonads. The data provided represent the estimation of soluble phosphate liberated from tricalcuim phosphate subtrate and reduction in pH of the medium due to microbial phosphate solubilization. Click here for file

Additional file 3 Antifungal activity of phosphate solubilizing fluorescent pseudomonads. The data provided represent the broad-spectrum antagonism towards important phytopathogenic fungi tested and possible antifungal potential of fluorescent pseudomonads. Click here for file
