# Supplementary material for: Assessment of genetic and functional diversity of phosphate solubilizing fluorescent pseudomonads isolated from rhizospheric soil
Source: BMC Microbiol. 2008 Dec 20;8:230. doi: 10.1186/1471-2180-8-230 (PMC2625360; doi:10.1186/1471-2180-8-230)
Supplement: Additional file 1 — Biochemical characterization and taxonomic identification of 80 phosphate solubilizing fluorescent pseudomonads. The data provided represent the taxonomic identification of strains on the basis of 16S rRNA nucleotide sequence based phylogenetic analysis, plant growth promoting traits (production of IAA, ACC deaminase and protease), plant growth affecting traits (production of pectinase and cellulase), biocontrol traits (production of chitinase, HCN and antagonism towards phytopathogenic fungi) and genotypic grouping on the basis of BOX-PCR fingerprint pattern. [file 1471-2180-8-230-S1.doc]

**Additional file 1.** Biochemical characterization and taxonomic identification of 80 phosphate solubilizing fluorescent pseudomonads

| Strain | *Pseudomonas* species | IAA | ACC | Prot | Chi | HCN | Pect | Cel | Ant | BOX cluster |
| --- | --- | --- | --- | --- | --- | --- | --- | --- | --- | --- |
| FPB9 | *P. aeruginosa*  (EF600807) | + | − | + | + | + | + | + | + | II |
| FPB15 | *P. aeruginosa*  (EF600813) | + | − | + | − | + | + | + | + | II |
| FPB16 | *P. aeruginosa*  (EF600814) | + | − | − | − | + | + | + | + | II |
| FPB17 | *P. aeruginosa*  (EF600815) | − | − | + | − | + | + | + | + | II |
| FPB49 | *P. aeruginosa* (EF600847) | + | − | + | − | − | − | + | − | II |
| FPB79 | *P. aeruginosa* (EF600877) | + | − | + | − | + | − | − | − | II |
| FPB80 | *P. aeruginosa* (EF600878) | + | − | + | − | + | − | − | − | II |
| Pw60 | *P. aeruginosa* (EF595661) | + | + | + | − | + | + | − | + | II |
| Pw61 | *P. aeruginosa*  (EF595662) | + | + | + | + | + | + | − | + | II |
| FP10 | *P. aeruginosa*  (DQ201401) | + | − | − | + | + | − | − | + | II |
| FP11 | *P. aeruginosa*  (DQ201402) | + | − | + | + | − | − | + | + | II |
| FPB18 | *P. aeruginsoa* (EF600816) | + | − | + | − | − | + | + | + | II |
| FP13 | *P. mosselli* (DQ201404) | + | − | + | − | − | − | − | + | II |
| FP7 | *P. mosselli* (DQ201398) | − | − | + | + | + | − | + | + | I |
| FPB4 | *P. monteilii* (EF600802) | − | − | − | − | − | − | − | − | III |
| FPB5 | *P. monteilii* (EF600803) | − | − | − | − | + | − | + | − | I |
| FPB21 | *P. monteilii*  (EF600819) | + | − | − | − | − | − | + | − | II |
| FPB22 | *P. monteilii*  (EF600820) | − | − | − | − | − | − | − | − | II |
| FPB23 | *P. monteilii*  (EF600821) | − | − | − | − | − | − | − | − | II |
| FPB24 | *P. monteilii* (EF600822) | − | − | − | − | − | − | − | − | II |

**Additional file 1.** Biochemical characterization and taxonomic identification of 80 phosphate solubilizing fluorescent pseudomonads (Contd.)

| Strain | *Pseudomonas* species | IAA | ACC | Prot | Chi | HCN | Pect | Cel | Ant | BOX cluster |
| --- | --- | --- | --- | --- | --- | --- | --- | --- | --- | --- |
| FPB27 | *P. monteilii*  (EF600825) | + | − | − | − | + | + | − | − | II |
| FPB33 | *P. monteilii* (EF600831) | − | − | + | − | − | − | − | − | II |
| FPB43 | *P. monteilii*  (EF600840) | + | − | − | − | − | − | − | − | II |
| FPB44 | *P. monteilii*  (EF600841) | + | − | − | − | + | − | − | − | II |
| FPB45 | *P. monteilii*  (EF600842) | + | − | − | − | + | − | − | − | II |
| FPB46 | *P. monteilii*  (EF600843) | + | + | − | − | − | + | + | − | II |
| FPB50 | *P. monteilii*  (EF600848) | + | − | − | − | − | + | + | + | II |
| FPB51 | *P. monteilii* (EF600849) | + | − | − | − | + | − | − | + | II |
| FPB52 | *P. monteilii*  (EF600850) | + | − | − | − | + | − | − | + | II |
| FPB53 | *P. monteilii*  (EF600851) | + | − | − | − | − | − | + | − | II |
| FPB54 | *P. monteilii*  (EF600852) | + | − | − | − | − | − | − | − | II |
| FPB55 | *P. monteilii*  (EF600853) | − | − | − | − | − | − | − | − | II |
| FPB56 | *P. monteilii*  (EF600854) | + | − | − | − | − | − | − | − | III |
| FPB58 | *P. monteilii*  (EF600856) | − | − | − | − | − | − | − | − | II |
| FPB59 | *P. monteilii*  (EF600857) | − | − | − | − | − | − | − | − | II |
| FPB63 | *P. monteilii*  (EF600861) | − | + | − | − | − | − | − | − | I |
| FPB73 | *P. monteilii*  (EF600871) | − | − | − | − | − | + | + | + | II |
| FPB74 | *P. monteilii*  (EF600872) | − | − | − | − | − | + | + | + | II |
| FPB75 | *P. monteilii*  (EF600873) | − | − | − | − | − | + | + | + | II |
| FPB77 | *P. monteilii*  (EF600875) | + | − | − | + | − | + | − | − | II |

**Additional file 1.** Biochemical characterization and taxonomic identification of 80 phosphate solubilizing fluorescent pseudomonads (Contd.)

| Strain | *Pseudomonas* species | IAA | ACC | Prot | Chi | HCN | Pect | Cel | Ant | BOX cluster |
| --- | --- | --- | --- | --- | --- | --- | --- | --- | --- | --- |
| FPB88 | *P. monteilii*  (EF600886) | + | − | − | − | − | − | − | − | II |
| FPB91 | *P. monteilii* (EF600889) | + | − | − | − | − | + | − | − | II |
| FPB94 | *P. monteilii*  (EF600892) | − | + | − | − | − | + | + | − | II |
| FPB95 | *P. monteilii*  (EF600893) | − | + | − | − | − | − | − | − | II |
| Pw102 | *P. monteilii* (EF595670) | − | + | + | − | − | + | − | − | II |
| Pw109 | *P. monteilii*  (EF595674) | − | − | + | − | − | + | − | − | II |
| Pw106 | *P. monteilii*  (EF595671) | + | + | − | − | − | + | − | − | II |
| FP15 | *P. monteilii* (DQ201406) | − | − | − | − | − | − | − | + | II |
| FP24 | *P. monteilii* (DQ201415) | − | + | + | − | − | − | − | + | II |
| FP25 | *P. monteilii* (DQ201416) | − | − | + | − | − | + | − | + | II |
| FPB25 | *P. plecoglo-ssicida* (EF600823) | + | − | − | − | − | − | − | − | II |
| FPB28 | *P. plecoglo-ssicida*  (EF600826) | + | − | − | − | − | − | − | − | II |
| FPB31 | *P. plecoglo-ssicida*  (EF600829) | + | − | − | − | − | − | − | − | II |
| FPB40 | *P. plecoglo-ssicida*  (EF600838) | + | − | − | − | − | + | + | + | II |
| FPB57 | *P. plecoglo-ssicida*  (EF600855) | + | − | − | − | − | − | − | − | II |
| FPB61 | *P. plecoglo-ssicida*  (EF600859) | + | + | + | − | − | + | + | − | II |
| FPB76 | *P. plecoglo-ssicida*  (EF600874) | − | − | − | − | − | − | + | − | II |

**Additional file 1.** Biochemical characterization and taxonomic identification of 80 phosphate solubilizing fluorescent pseudomonads (Contd.)

| Strain | *Pseudomonas* species | IAA | ACC | Prot | Chi | HCN | Pect | Cel | Ant | BOX cluster |
| --- | --- | --- | --- | --- | --- | --- | --- | --- | --- | --- |
| FPB86 | *P. plecoglo-ssicida*  (EF600884) | + | − | − | − | − | − | − | − | II |
| FPB93 | *P. plecoglo-ssicida*  (EF600891) | + | − | + | − | − | − | − | − | II |
| FP12 | *P. plecoglo-ssicida*  (DQ201403) | + | − | + | − | − | − | − | + | II |
| FPB26 | *P. putida* (EF600824) | + | − | − | − | − | − | − | − | II |
| FPB47 | *P. putida* (EF600845) | + | − | − | − | + | − | − | − | II |
| FPB85 | *P. putida* (EF600883) | − | − | − | − | − | + | + | − | I |
| Pw66 | *P. putida* (EF595663) | − | − | − | − | − | + | + | − | I |
| Pw68 | *P. putida* (EF595664) | − | − | − | − | − | + | + | − | I |
| Pw70 | *P. putida* (EF595665) | − | − | − | − | − | + | − | + | I |
| Pw71 | *P. putida* (EF595666) | − | + | − | + | − | + | − | + | I |
| Pw72 | *P. putida* (EF595667) | − | − | − | − | − | + | − | + | I |
| Pw81 | *P. putida* (EF595669) | − | + | − | − | + | + | − | − | I |
| Pw107 | *P. putida* (EF595672) | − | + | − | − | − | + | − | − | I |
| Pw108 | *P. putida* (EF595673) | − | − | − | − | − | + | − | − | I |
| FPB92 | *P. putida* (EF600890) | − | − | − | − | − | − | − | − | I |
| FP23 | *P. fulva*  (DQ201414) | − | − | − | − | − | − | − | + | I |
| FP2 | *P. fluorescens*  (DQ201393 ) | − | − | − | − | − | − | − | + | I |
| FP3 | *P. fluorescens*  (DQ201394) | − | − | + | − | − | + | − | + | I |
| FP5 | *P. fluorescens*  (DQ201396) | − | − | − | − | + | + | − | + | I |

**Additional file 1.** Biochemical characterization and taxonomic identification of 80 phosphate solubilizing fluorescent pseudomonads (Contd.)

| Strain | *Pseudomonas* species | IAA | ACC | Prot | Chi | HCN | Pect | Cel | Ant | BOX cluster |
| --- | --- | --- | --- | --- | --- | --- | --- | --- | --- | --- |
| FP9 | *P. fluorescens*  (DQ201400) | − | − | + | − | − | − | − | + | I |
| FP14 | *P. fluorescens*  (DQ201405) | − | − | − | − | + | − | − | + | I |
| FP16 | *P. fluorescens*  (DQ201407) | − | − | − | − | − | − | + | + | I |
| FP17 | *P. fluorescens*  (DQ201408) | − | − | − | − | − | − | + | + | I |

IAA, indole-3-acetic acid; ACC, aminocyclopropane-1-carboxylate deaminase; Prot, protease; HCN, hydrogen cyanide; Pect, pectinase; Cel, cellulase; Ant, antifungal activity. GenBank accession numbers were provided in the paranthesis.
