# Supplementary material for: Assessment of genetic and functional diversity of phosphate solubilizing fluorescent pseudomonads isolated from rhizospheric soil
Source: BMC Microbiol. 2008 Dec 20;8:230. doi: 10.1186/1471-2180-8-230 (PMC2625360; doi:10.1186/1471-2180-8-230)
Supplement: Additional file 2 — Tricalcium phosphate solubilization by the strains of fluorescent pseudomonads. The data provided represent the estimation of soluble phosphate liberated from tricalcuim phosphate subtrate and reduction in pH of the medium due to microbial phosphate solubilization. [file 1471-2180-8-230-S2.doc]

## Additional file 2. Tricalcium phosphate solubilization by the strains of fluorescent pseudomonads

| Strain | Days | | | | | | | | | | | | | | | | |  |
| --- | --- | --- | --- | --- | --- | --- | --- | --- | --- | --- | --- | --- | --- | --- | --- | --- | --- | --- |
|  | 1 | |  | 3 | |  | | 5 | |  | | 7 | |  | 10 | |  | |
|  | P solubilized ^#^ | pH | | P solubilized | pH | | P solubilized | | pH | | P solubilized | | pH | | P solubilized | pH | |  |
| FPB4 | 8.08 ± 0.63^cd^ | 6.5 | | 23.57 ± 0.10^d^ | 6.3 | | 39.38 ± 0.71^d^ | | 5.9 | | 47.53 ± 0.64^d^ | | 5.6 | | 60.57 ± 0.87^d^ | 5.3 | |  |
| FPB5 | 4.62 ± 0.93^ab^ | 6.6 | | 27.58 ± 0.34^e^ | 6.2 | | 51.43 ± 0.45^g^ | | 5.7 | | 67.58 ± 0.19^gh^ | | 5.3 | | 78.53 ± 0.73^g^ | 4.9 | |  |
| FPB9 | 4.89 ± 0.63^b^ | 6.7 | | 24.54 ± 0.10^de^ | 6.3 | | 30.27 ± 0.16^c^ | | 6.1 | | 32.52 ± 0.03^bc^ | | 6.0 | | 33.34 ± 0.93^ab^ | 5.9 | |  |
| FPB15 | 6.30 ± 0.61^b^ | 6.6 | | 29.35 ± 0.30^f^ | 6.1 | | 45.35 ± 0.21^f^ | | 5.8 | | 58.13 ± 0.19^f^ | | 5.3 | | 68.02 ± 0.40^e^ | 5.0 | |  |
| FPB16 | 8.84 ± 1.06^d^ | 6.5 | | 31.26 ± 0.77^fg^ | 6.3 | | 40.87 ± 0.12^e^ | | 5.9 | | 48.70 ± 0.50^de^ | | 5.6 | | 58.68 ± 0.68^d^ | 5.4 | |  |
| FPB17 | 6.72 ± 0.58^bc^ | 6.5 | | 28.05 ± 0.15^ef^ | 6.1 | | 46.46 ± 0.12^f^ | | 5.8 | | 61.66 ± 0.68^fg^ | | 5.3 | | 75.41 ± 1.05^fg^ | 5.0 | |  |
| FPB49 | 18.80 ± 0.87^g^ | 6.3 | | 36.55 ± 0.09^g^ | 5.9 | | 46.60 ± 0.75^f^ | | 5.7 | | 67.87 ± 0.81^gh^ | | 5.3 | | 82.80 ± 0.14^h^ | 4.9 | |  |
| FPB79 | 5.52 ± 0.59^b^ | 6.6 | | 20.70 ± 0.69^cd^ | 6.2 | | 30.34 ± 2.12^c^ | | 6.0 | | 39.15 ± 0.07^c^ | | 5.8 | | 51.10 ± 0.21^c^ | 5.6 | |  |
| FPB80 | 16.90 ± 0.61^g^ | 6.4 | | 50.40 ± 1.02^h^ | 5.7 | | 75.79 ± 0.75^i^ | | 5.3 | | 91.22 ± 1.73^k^ | | 4.9 | | 100.0 ± 1.06^j^ | 4.5 | |  |
| Pw60 | 10.40 ± 0.46^de^ | 6.4 | | 34.74 ± 0.75^g^ | 6.0 | | 53.15 ± 0.07^gh^ | | 5.8 | | 73.37 ± 0.15^hi^ | | 5.3 | | 85.23 ± 0.33^hi^ | 4.9 | |  |
| Pw61 | 4.41 ± 0.19^a^ | 6.6 | | 25.36 ± 0.03^e^ | 6.1 | | 40.50 ± 0.45^e^ | | 5.9 | | 57.81 ± 0.35^f^ | | 5.4 | | 75.87 ± 0.76^fg^ | 5.0 | |  |
| FP10 | 5.50 ± 0.09^b^ | 6.6 | | 25.42 ± 0.70^e^ | 6.2 | | 39.03 ± 0.10^d^ | | 5.9 | | 49.27 ± 0.52^de^ | | 5.5 | | 61.97 ± 0.40^d^ | 5.3 | |  |
| FP11 | 5.08 ± 1.08^b^ | 6.6 | | 19.92 ± 0.53^c^ | 6.1 | | 30.31 ± 0.51^c^ | | 5.9 | | 38.37 ± 0.23^c^ | | 5.7 | | 45.91 ± 0.27^bc^ | 5.5 | |  |
| FPB18 | 15.07 ± 0.42^f^ | 6.4 | | 34.24 ± 0.11^g^ | 5.9 | | 59.73 ± 2.97^hi^ | | 5.4 | | 75.22 ± 0.87^i^ | | 4.9 | | 88.25 ± 0.19^i^ | 4.3 | |  |
| FP7 | 6.80 ± 0.93^c^ | 6.6 | | 29.45 ± 0.94^f^ | 6.2 | | 51.77 ± 0.27^g^ | | 5.9 | | 75.68 ± 0.62^ij^ | | 5.6 | | 90.52 ± 0.71^i^ | 5.3 | |  |
| FP13 | 4.55 ± 0.95^ab^ | 6.6 | | 21.08 ± 0.69^d^ | 6.2 | | 34.93 ± 0.57^cd^ | | 5.9 | | 47.91 ± 0.4^d^ | | 5.6 | | 63.17 ± 0.45^de^ | 5.3 | |  |
| FPB21 | 7.50 ± 0.37^c^ | 6.9 | | 15.05 ± 0.30^bc^ | 6.2 | | 34.14 ± 0.28^cd^ | | 6.0 | | 53.03 ± 3.35^ef^ | | 5.6 | | 76.86 ± 0.35^g^ | 5.1 | |  |
| FPB22 | 4.56 ± 0.60^ab^ | 6.6 | | 14.06 ± 0.33^b^ | 6.4 | | 39.70 ± 0.28^de^ | | 5.9 | | 63.84 ± 3.3^g^ | | 5.5 | | 78.45 ± 0.26^g^ | 5.0 | |  |
| FPB23 | 9.22 ± 0.14^d^ | 6.5 | | 11.48 ± 0.14^a^ | 6.3 | | 25.17 ± 0.03^b^ | | 6.0 | | 38.73 ± 0.29^c^ | | 5.8 | | 45.41 ± 0.26^bc^ | 5.7 | |  |
| FPB24 | 3.30 ± 0.56^a^ | 6.8 | | 13.54 ± 0.03^b^ | 6.3 | | 40.91 ± 1.62^e^ | | 6.0 | | 47.74 ± 1.79^d^ | | 5.6 | | 67.47 ± 0.10^e^ | 5.1 | |  |
| FPB27 | 9.99 ± 0.38^d^ | 6.6 | | 21.25 ± 0.29^d^ | 6.1 | | 38.12 ± 0.27^d^ | | 5.8 | | 52.34 ± 0.64^e^ | | 5.6 | | 63.59 ± 0.12^de^ | 5.3 | |  |
| FPB33 | 15.47 ± 0.50^fg^ | 6.4 | | 28.53 ± 0.28^ef^ | 6.0 | | 47.70 ± 0.32^fg^ | | 5.4 | | 62.05 ± 0.90^g^ | | 5.1 | | 77.61 ± 0.29^g^ | 4.9 | |  |
| FPB43 | 4.76 ± 0.24^b^ | 6.7 | | 25.76 ± 0.93^e^ | 6.1 | | 56.28 ± 0.43^h^ | | 5.5 | | 70.52 ± 0.37^h^ | | 5.1 | | 84.06 ± 0.52^h^ | 4.8 | |  |
| FPB44 | 7.35 ± 0.63^c^ | 6.7 | | 16.48 ± 0.15^bc^ | 6.4 | | 39.09 ± 0.78^d^ | | 5.8 | | 56.49 ± 0.21^f^ | | 5.4 | | 71.86 ± 0.63^f^ | 5.1 | |  |
| FPB45 | 9.59 ± 0.05^d^ | 6.4 | | 29.05 ± 1.37^f^ | 6.0 | | 43.87 ± 2.87^ef^ | | 5.6 | | 64.58 ± 0.22^g^ | | 5.3 | | 73.35 ± 0.30^f^ | 5.0 | |  |
| FPB46 | 5.46 ± 0.73^b^ | 6.7 | | 25.30 ± 0.36^e^ | 6.1 | | 32.43 ± 3.07^c^ | | 5.9 | | 54.35 ± 0.35^ef^ | | 5.5 | | 62.06 ± 5.60^de^ | 5.3 | |  |
| FPB50 | 7.26 ± 1.27^c^ | 6.8 | | 12.49 ± 0.38^ab^ | 6.5 | | 19.21 ± 0.83^a^ | | 6.1 | | 22.15 ± 0.28^a^ | | 6.0 | | 29.45 ± 0.26^a^ | 5.9 | |  |
| FPB51 | 11.59 ± 0.28^e^ | 6.5 | | 32.46 ± 0.76^fg^ | 6.0 | | 58.30 ± 0.24^h^ | | 5.3 | | 75.36 ± 0.75^i^ | | 4.9 | | 92.75 ± 0.49^ij^ | 4.5 | |  |
| FPB52 | 2.52 ± 1.15^a^ | 6.9 | | 24.02 ± 0.48^de^ | 6.2 | | 34.26 ± 0.21^cd^ | | 5.8 | | 50.19 ± 1.89^de^ | | 5.5 | | 59.96 ± 0.35^d^ | 5.2 | |  |
| FPB53 | 5.10 ± 0.52^b^ | 6.7 | | 18.70 ± 0.18^c^ | 6.4 | | 39.80 ± 2.23^de^ | | 5.8 | | 48.54 ± 1.99^de^ | | 5.4 | | 67.03 ± 0.05^e^ | 5.2 | |  |
| FPB54 | 5.84 ± 0.37^b^ | 6.6 | | 28.59 ± 0.13^ef^ | 6.1 | | 51.05 ± 0.70^g^ | | 5.5 | | 72.91 ± 0.57^hi^ | | 5.3 | | 87.83 ± 0.49^hi^ | 4.6 | |  |

## Additional file 2. Tricalcium phosphate solubilization by the strains of fluorescent pseudomonads (Contd.)

| Strain | Days | | | | | | | | | | | | | | | | |  |
| --- | --- | --- | --- | --- | --- | --- | --- | --- | --- | --- | --- | --- | --- | --- | --- | --- | --- | --- |
|  | 1 | |  | 3 | |  | | 5 | |  | | 7 | |  | 10 | |  | |
|  | P solubilized | pH | | P solubilized | pH | | P solubilized | | pH | | P solubilized | | pH | | P solubilized | pH | |  |
| FPB55 | 11.13 ± 1.21^e^ | 6.6 | | 29.68 ± 0.61^f^ | 6.1 | | 50.32 ± 1.21^g^ | | 5.5 | | 69.30 ± 0.03^h^ | | 5.3 | | 85.51 ± 1.29^hi^ | 4.6 | |  |
| FPB56 | 4.20 ± 0.34^a^ | 6.9 | | 12.99 ± 0.19^b^ | 6.4 | | 31.03 ± 1.48^c^ | | 5.9 | | 51.71 ± 0.35^e^ | | 5.5 | | 70.28 ± 0.73^ef^ | 5.2 | |  |
| FPB58 | 10.69 ± 1.43^de^ | 6.5 | | 25.70 ± 0.07^e^ | 6.1 | | 46.31 ± 1.68^f^ | | 5.6 | | 57.44 ± 0.35^f^ | | 5.3 | | 74.57 ± 0.38^fg^ | 5.0 | |  |
| FPB59 | 8.96 ± 0.30^d^ | 6.6 | | 23.60 ± 0.12^d^ | 6.2 | | 41.71 ± 1.81^ef^ | | 5.7 | | 61.15 ± 0.78^fg^ | | 5.4 | | 74.44 ± 0.22^f^ | 5.1 | |  |
| FPB63 | 4.97 ± 0.19^b^ | 6.7 | | 20.32 ± 0.40^cd^ | 6.3 | | 28.05 ± 0.18^bc^ | | 6.0 | | 40.18 ± 0.42^cd^ | | 5.7 | | 53.93 ± 0.56^c^ | 5.5 | |  |
| FPB73 | 9.30 ± 0.32^d^ | 6.5 | | 23.97 ± 0.30^d^ | 6.2 | | 47.49 ± 0.66^fg^ | | 5.7 | | 62.85 ± 1.34^g^ | | 5.2 | | 79.90 ± 0.22^gh^ | 4.9 | |  |
| FPB74 | 6.40 ± 0.42^bc^ | 6.8 | | 26.43 ± 0.21^e^ | 6.2 | | 46.37 ± 0.27^f^ | | 5.5 | | 71.04 ± 0.45^h^ | | 5.0 | | 85.04 ± 0.30^hi^ | 4.4 | |  |
| FPB75 | 4.14 ± 0.77^a^ | 6.8 | | 20.03 ± 0.31^cd^ | 6.2 | | 39.63 ± 0.22^de^ | | 5.7 | | 59.75 ± 0.28^f^ | | 5.4 | | 68.06 ± 0.25^e^ | 5.2 | |  |
| FPB77 | 3.99 ± 0.33^a^ | 6.9 | | 9.41 ± 0.33^a^ | 6.6 | | 20.53 ± 0.88^ab^ | | 6.1 | | 30.92 ± 0.28^bc^ | | 6.0 | | 39.70 ± 0.35^b^ | 5.7 | |  |
| FPB88 | 8.31 ± 1.03^d^ | 6.6 | | 20.20 ± 0.40^cd^ | 6.2 | | 39.43 ± 2.08^d^ | | 5.8 | | 60.92 ± 0.43^fg^ | | 5.4 | | 72.55 ± 0.35^f^ | 5.1 | |  |
| FPB91 | 6.97 ± 0.07^c^ | 6.8 | | 20.17 ± 0.75^cd^ | 6.2 | | 36.46 ± 1.52^d^ | | 5.7 | | 56.58 ± 0.27^f^ | | 5.3 | | 67.66 ± 0.51^e^ | 5.2 | |  |
| FPB94 | 7.29 ± 0.35^c^ | 6.6 | | 11.76 ± 0.03^a^ | 6.4 | | 25.11 ± 0.03^b^ | | 6.2 | | 45.01 ± 0.62^d^ | | 5.7 | | 57.33 ± 0.35^cd^ | 5.3 | |  |
| FPB95 | 11.48 ± 0.63^e^ | 6.7 | | 16.69 ± 0.10^c^ | 6.4 | | 26.11 ± 0.68^b^ | | 6.0 | | 42.57 ± 0.43^cd^ | | 5.7 | | 62.12 ± 0.30^de^ | 5.3 | |  |
| Pw102 | 2.92 ± 0.51^a^ | 6.9 | | 11.92 ± 0.25^a^ | 6.5 | | 25.26 ± 0.12^b^ | | 6.1 | | 45.34 ± 1.41^d^ | | 5.7 | | 68.02 ± 0.52^e^ | 5.0 | |  |
| Pw106 | 4.68 ± 0.24^ab^ | 6.8 | | 18.52 ± 0.15^c^ | 6.3 | | 31.74 ± 0.41^c^ | | 5.9 | | 45.47 ± 0.22^d^ | | 5.7 | | 56.85 ± 0.52^cd^ | 5.3 | |  |
| Pw109 | 8.36 ± 0.56^d^ | 6.7 | | 23.53 ± 0.32^d^ | 6.2 | | 48.18 ± 1.11^fg^ | | 5.7 | | 58.45 ± 0.22^f^ | | 5.2 | | 79.10 ± 0.41^gh^ | 4.9 | |  |
| FP15 | 4.89 ± 0.07^b^ | 6.7 | | 23.18 ± 0.80^d^ | 6.3 | | 42.95 ± 0.68^ef^ | | 5.9 | | 57.75 ± 0.20^f^ | | 5.4 | | 71.33 ± 1.00^f^ | 5.2 | |  |
| FP24 | 5.63 ± 0.69^b^ | 6.7 | | 23.28 ± 0.05^d^ | 6.2 | | 38.90 ± 0.08^d^ | | 5.9 | | 52.06 ± 0.10^e^ | | 5.6 | | 76.33 ± 0.70^g^ | 5.2 | |  |
| FP25 | 4.39 ± 0.68^a^ | 6.8 | | 24.67 ± 0.15^e^ | 6.3 | | 46.10 ± 0.11^f^ | | 5.9 | | 61.84 ± 0.07^fg^ | | 5.3 | | 74.08 ± 0.69^f^ | 5.0 | |  |
| FPB25 | 10.22 ± 0.87^d^ | 6.7 | | 24.96 ± 0.03^e^ | 6.3 | | 44.48 ± 0.46^f^ | | 5.7 | | 58.89 ± 0.21^f^ | | 5.3 | | 72.38 ± 1.69^f^ | 5.0 | |  |
| FPB28 | 4.20 ± 0.84^a^ | 6.9 | | 22.61 ± 0.12^d^ | 6.1 | | 50.85 ± 2.58^g^ | | 5.7 | | 68.94 ± 0.61^h^ | | 5.2 | | 81.70 ± 0.74^gh^ | 4.9 | |  |
| FPB31 | 15.43 ± 0.34^f^ | 6.4 | | 34.14 ± 0.28^g^ | 5.8 | | 58.36 ± 0.27^h^ | | 5.3 | | 75.93 ± 0.26^ij^ | | 4.9 | | 86.64 ± 0.75^hi^ | 4.6 | |  |
| FPB40 | 13.60 ± 0.63^ef^ | 6.6 | | 24.98 ± 0.81^e^ | 6.2 | | 44.38 ± 0.64^f^ | | 5.7 | | 58.57 ± 0.14^f^ | | 5.4 | | 71.88 ± 0.77^f^ | 5.0 | |  |
| FBP57 | 5.12 ± 0.33^b^ | 6.7 | | 27.08 ± 0.52^e^ | 6.3 | | 47.32 ± 0.17^fg^ | | 5.6 | | 65.20 ± 0.19^g^ | | 5.2 | | 79.27 ± 1.66^gh^ | 4.9 | |  |
| FBP61 | 4.26 ± 0.29^a^ | 6.8 | | 18.50 ± 0.09^c^ | 6.3 | | 37.37 ± 0.10^d^ | | 5.9 | | 48.24 ± 0.51^de^ | | 5.5 | | 62.48 ± 0.33^de^ | 5.2 | |  |
| FPB76 | 10.08 ± 0.89^d^ | 6.6 | | 34.18 ± 0.10^g^ | 5.9 | | 39.38 ± 0.33^d^ | | 5.7 | | 52.04 ± 0.50^e^ | | 5.4 | | 67.45 ± 2.75^e^ | 5.1 | |  |
| FBP86 | 14.11 ± 1.58^f^ | 6.4 | | 27.65 ± 0.28^e^ | 6.0 | | 46.40 ± 0.41^f^ | | 5.6 | | 56.30 ± 0.39^f^ | | 5.3 | | 66.97 ± 2.21^e^ | 5.1 | |  |
| FPB93 | 9.40 ± 0.16^d^ | 6.6 | | 23.76 ± 0.38^d^ | 6.0 | | 40.01 ± 0.47^e^ | | 5.8 | | 48.87 ± 1.38^de^ | | 5.7 | | 66.80 ± 0.90^e^ | 5.1 | |  |
| FP12 | 5.25 ± 0.71^b^ | 6.5 | | 26.70 ± 0.53^e^ | 6.1 | | 44.21 ± 0.47^f^ | | 5.8 | | 56.79 ± 0.31^f^ | | 5.3 | | 74.32 ± 0.87^f^ | 5.0 | |  |
| FPB26 | 10.33 ± 0.16^de^ | 6.6 | | 29.14 ± 0.40^f^ | 5.9 | | 48.03 ± 0.26^fg^ | | 5.6 | | 64.43 ± 0.81^g^ | | 5.2 | | 75.55 ± 1.07^fg^ | 4.9 | |  |
| FBP47 | 6.97 ± 0.66^c^ | 6.5 | | 27.02 ± 0.41^e^ | 6.1 | | 46.33 ± 1.37^f^ | | 5.8 | | 68.16 ± 3.41^h^ | | 5.2 | | 82.40 ± 0.68^h^ | 4.9 | |  |

## Additional file 2. Tricalcium phosphate solubilization by the strains of fluorescent pseudomonads (Contd.)

| Strain | Days | | | | | | | | | | | | | | | | |  |
| --- | --- | --- | --- | --- | --- | --- | --- | --- | --- | --- | --- | --- | --- | --- | --- | --- | --- | --- |
|  | 1 | |  | 3 | |  | | 5 | |  | | 7 | |  | 10 | |  | |
|  | P solubilized | pH | | P solubilized | pH | | P solubilized | | pH | | P solubilized | | pH | | P solubilized | pH | |  |
| FBP85 | 12.55 ± 0.43^ef^ | 6.5 | | 31.17 ± 0.41^fg^ | 5.9 | | 52.27 ± 0.12^g^ | | 5.6 | | 69.99 ± 0.59^h^ | | 5.1 | | 82.04 ± 0.33^h^ | 4.9 | |  |
| FPB92 | 6.53 ± 0.63^bc^ | 6.4 | | 22.94 ± 0.03^d^ | 6.1 | | 40.66 ± 0.15^e^ | | 5.8 | | 56.79 ± 0.54^f^ | | 5.6 | | 70.66 ± 1.92^ef^ | 5.1 | |  |
| Pw66 | 4.55 ± 0.33^ab^ | 6.8 | | 17.44 ± 0.19^c^ | 6.3 | | 35.52 ± 0.33^d^ | | 5.9 | | 53.45 ± 0.45^ef^ | | 5.4 | | 65.90 ± 0.63^e^ | 5.2 | |  |
| Pw68 | 5.35 ± 0.23^b^ | 6.7 | | 10.75 ± 0.19^a^ | 6.4 | | 20.26 ± 0.35^ab^ | | 6.1 | | 26.47 ± 0.73^a^ | | 5.9 | | 30.92 ± 0.30^a^ | 5.7 | |  |
| Pw70 | 9.70 ± 0.68^d^ | 6.6 | | 35.58 ± 0.99^g^ | 6.0 | | 58.47 ± 1.17^h^ | | 5.4 | | 74.86 ± 0.97^i^ | | 5.1 | | 92.87 ± 0.62^ij^ | 4.7 | |  |
| Pw71 | 6.03 ± 0.54^b^ | 6.8 | | 23.07 ± 0.45^d^ | 6.2 | | 44.23 ± 0.51^f^ | | 6.0 | | 58.24 ± 1.11^f^ | | 5.8 | | 77.82 ± 0.49^g^ | 5.3 | |  |
| Pw72 | 8.21 ± 1.66^cd^ | 6.5 | | 23.07 ± 0.45^d^ | 6.1 | | 44.23 ± 0.51^f^ | | 5.8 | | 58.24 ± 1.11^f^ | | 5.5 | | 77.82 ± 0.49^g^ | 5.1 | |  |
| Pw81 | 4.35 ± 0.09^a^ | 6.8 | | 9.76 ± 0.30^a^ | 6.4 | | 18.41 ± 0.12^a^ | | 6.0 | | 24.44 ± 0.28^a^ | | 5.9 | | 32.67 ± 0.30^ab^ | 5.7 | |  |
| Pw107 | 5.02 ± 0.80^b^ | 6.5 | | 30.33 ± 0.67^f^ | 5.9 | | 53.45 ± 0.40^gh^ | | 5.7 | | 64.45 ± 0.09^g^ | | 5.3 | | 82.25 ± 2.02^h^ | 4.8 | |  |
| Pw108 | 4.95 ± 0.30^b^ | 6.8 | | 20.07 ± 0.59^cd^ | 6.2 | | 28.70 ± 0.38^bc^ | | 5.9 | | 48.05 ± 0.31^de^ | | 5.5 | | 60.21 ± 0.52^d^ | 5.2 | |  |
| FP23 | 8.65 ± 1.07^d^ | 6.4 | | 26.70 ± 0.19^e^ | 6.1 | | 49.50 ± 0.37^g^ | | 5.7 | | 69.13 ± 1.13^h^ | | 5.3 | | 80.93 ± 1.05^gh^ | 4.9 | |  |
| FP2 | 11.44 ± 1.11^e^ | 6.3 | | 37.89 ± 0.83^g^ | 5.9 | | 58.24 ± 0.82^h^ | | 5.5 | | 80.32 ± 0.46^j^ | | 5.0 | | 103.2 ± 0.80^j^ | 4.4 | |  |
| FP3 | 14.21 ± 1.76^f^ | 6.3 | | 43.83 ± 0.25^h^ | 6.0 | | 66.19 ± 1.08^i^ | | 5.3 | | 90.33 ± 0.28^k^ | | 4.8 | | 99.65 ± 0.58^j^ | 4.5 | |  |
| FP5 | 10.08 ± 0.95^d^ | 6.5 | | 38.82 ± 0.15^gh^ | 5.9 | | 66.57 ± 0.42^i^ | | 5.3 | | 81.12 ± 0.84^jk^ | | 5.0 | | 105.3 ± 0.49^j^ | 4.4 | |  |
| FP9 | 6.99 ± 0.94^c^ | 6.6 | | 38.58 ± 0.42^gh^ | 6.0 | | 58.57 ± 0.72^h^ | | 5.3 | | 80.42 ± 2.85^j^ | | 4.9 | | 94.87 ± 1.51^ij^ | 4.5 | |  |
| FP14 | 9.05 ± 1.58^d^ | 6.5 | | 40.37 ± 0.44^h^ | 6.0 | | 57.31 ± 0.77^h^ | | 5.7 | | 83.28 ± 0.42^jk^ | | 4.9 | | 99.97 ± 1.33^j^ | 4.5 | |  |
| FP16 | 5.16 ± 0.42^b^ | 6.7 | | 18.79 ± 0.16^c^ | 6.3 | | 40.71 ± 0.24^e^ | | 5.8 | | 66.00 ± 0.05^gh^ | | 5.4 | | 74.63 ± 0.29^fg^ | 5.1 | |  |
| FP17 | 15.53 ± 1.34^fg^ | 6.4 | | 36.34 ± 1.05^g^ | 5.9 | | 59.72 ± 0.56^hi^ | | 5.4 | | 76.25 ± 0.37^ij^ | | 5.1 | | 89.91 ± 0.67^i^ | 4.8 | |  |

Mean values with in the column followed by different superscript letters are significantly different according to Duncan’s multiple range test (*p <* 0.05). Data represents the average of three replications. ^#^ Phosphate solubilized in μg ml^-1^ ± S.E.
