# Supplementary material for: Assessment of genetic and functional diversity of phosphate solubilizing fluorescent pseudomonads isolated from rhizospheric soil
Source: BMC Microbiol. 2008 Dec 20;8:230. doi: 10.1186/1471-2180-8-230 (PMC2625360; doi:10.1186/1471-2180-8-230)
Supplement: Additional file 3 — Antifungal activity of phosphate solubilizing fluorescent pseudomonads. The data provided represent the broad-spectrum antagonism towards important phytopathogenic fungi tested and possible antifungal potential of fluorescent pseudomonads. [file 1471-2180-8-230-S3.doc]

## Additional file 3. Antifungal activity of phosphate solubilizing fluorescent pseudomonads

| Species | Strain | Fungi inhibited | Antifungal potential |
| --- | --- | --- | --- |
| *P. aeruginosa* | FPB9 | *Cy.f., Cy.s., S.o., P.o., R.s.,B.c., M.p.,P.t., C.f., C.c.,C.g.* | HCN, CHI |
|  | FPB15 | *Cy.f., Cy.s., P.o., R.s., B.c.,* *M.p., P.t., C.f., C.c., C.g.* | HCN, PRN |
|  | FPB16 | *Cy.f., Cy.s., P.o., R.s., B.c.,* *M.p., P.t., C.f., C.c., C.g.* | HCN, DAPG, PRN |
|  | FPB17 | *Cy.f., Cy.s., S.o., P.o., R.s.,B.c., M.p., P.t., C.f., C.c., C.g.* | HCN, DAPG |
|  | FP10 | *Cy.f., Cy.s., S.o., R.s., B.c., M.p., P.t., C.f.,C.c.,C.g., M.g.,F.o.c.,F.o.v.* | HCN, CHI, DAPG |
|  | FP11 | *Cy.f., Cy.s., S.o., R.s., B.c., M.p., P.t., C.f.,C.c.,C.g., M.g.,F.o.c.,F.o.v.* | CHI |
|  | FPB18 | *Cy.f., Cy.s., P.o., R.s., B.c.,* *M.p., P.t., C.f., C.c., C.g.* | PCA |
|  | Pw60 | *Cy.s., S.o., R.s.,* *M.p., C.g.,* *F.* sp. | HCN, PCA |
|  | Pw61 | *Cy.s., S.o., R.s.,* *M.p., C.g.,* *F.* sp. | HCN, PCA, CHI |
| *P. mosselii* | FP7 | *S.o., R.s., M.p., P.t., C.f., C.c., C.g., M.g., F.o.c., F.o.v.,B.c.* | HCN, CHI, DAPG, PCA, PRN, PLT |
|  | FP13 | *Cy.f., Cy.s., S.o.,R.s., B.c., M.p., P.t., C.f., C.c.,C.g.,M.g.,F.o.c.,F.o.v.* | ND |
| *P. monteilii* | FPB50 | *S.o, M.p.* | ND |
|  | FPB51 | *Cy.f.,Cys., S.o.,P.o., R.s.,* *B.c., M.p., P.t., C.f., C.c.,C.g.* | HCN, DAPG |
|  | FPB52 | *Cy.f.,Cy.s., S.o., P.o.,* *R.s., B.c., M.p., P.t., C.f., C.c.,C.g.* | HCN, PCA |
|  | FPB73 | *S.o., M.p.* | ND |
|  | FPB74 | *S.o., M.p., P.t.* | PRN |
|  | FPB75 | *Cy.f., Cy.s., S.o., P.o., B.c., M.p., P.t.* | PRN, PCA |
|  | FP15 | *M.p., P.t., C.f., C.c., C.g.* | PRN, PCA |
|  | FP24 | *Cy.f., Cy.s., S.o., R.s., B.c., M.p., P.t., C.f., C.c.,C.g., M.g.,F.o.c.,F.o.v.* | ND |
|  | FP25 | *Cy.f., Cy.s., S.o., R.s., B.c., M.p., P.t., C.f., C.c.,C.g., M.g.,F.o.c.,* *F.o.v.* | ND |
| *P. plecoglossicida* | FPB40 | *S.o., R.s., P.t., C.f.* | ND |
| *P. putida* | Pw70 | *S.o.* | DAPG |
|  | Pw71 | *S.o.* | DAPG, CHI |
|  | Pw72 | *S.o.* | DAPG |
|  | FP12 | *Cy.f., Cy.s., S.o., R.s., B.c., M.p., P.t., C.f., C.c.,C.g., M.g.,F.o.c., F.o.v.* | ND |
| *P. fulva* | FP23 | *M.p.* | ND |
| *P. fluorescens* | FP2 | *R.s., P.t.* | DAPG, PRN, PLT |
|  | FP3 | *R.s., P.t.* | DAPG, PRN, PLT |
|  | FP5 | *B.c.* | HCN, DAPG, PRN, PLT |
|  | FP9 | *S.o.* | PLT |
|  | FP14 | *S.o., M.p., C.g.* | HCN, DAPG, PRN, PLT |
|  | FP16 | *M.p., C.c.* | ND |
|  | FP17 | *M.p., P.t., C.c., M.g.* | ND |

*Cy.f., Cylindrocladium floridanum; Cy.s., Cy. scoparium; S.o., Sarocladium oryzae; P.o., Pyricularia oryzae; R.s., Rhizoctonia solani; B.c., Botrytis cinerea; M.p., Macrophomina phaseolina; P.t., Pestalotia theae; C.f., Colletotrichum falcatum; C.c., C. capsici; C.g., C. gleosporoides; M.g., Magnaporthe grisea;F.o.c., Fusarium oxysporum* f.sp*. cubense; F.o.v., Fusarium oxysporum* f.sp*. vasinfectum;* HCN, hydrogen cyanide; CHI, chitinase; DAPG, 2,4-diacetyl phloroglucinol; PRN, pyrrolnitrin; PLT, pyoluteorin; PCA, phenazine-1-carboxylic acid; ND, Not determined.
